# Hyperspectral imaging combined with CNN for maize variety identification

**DOI:** 10.3389/fpls.2023.1254548

**Published:** 2023-09-08

**Authors:** Fu Zhang, Fangyuan Zhang, Shunqing Wang, Lantao Li, Qiang Lv, Sanling Fu, Xinyue Wang, Qingfeng Lv, Yakun Zhang

**Affiliations:** ^1^ College of Agricultural Equipment Engineering, Henan University of Science and Technology, Luoyang, China; ^2^ Collaborative Innovation Center of Machinery Equipment Advanced Manufacturing of Henan Province, Henan University of Science and Technology, Luoyang, China; ^3^ College of Resources and Environment, Henan Agricultural University, Zhengzhou, China; ^4^ College of Agriculture / Peon, Henan University of Science and Technology, Luoyang, China; ^5^ College of Physical Engineering, Henan University of Science and Technology, Luoyang, China

**Keywords:** hyperspectral imaging technology, maize, high dimensional feature mapping, convolution neural network, non-destructive testing

## Abstract

**Introduction:**

As the third largest food crop in the world, maize has wide varieties with similar appearances, which makes identification difficult. To solve the problem of identification of hybrid maize varieties, a method based on hyperspectral image technology combined with a convolutional neural network (CNN) is proposed to identify maize varieties.

**Methods:**

In this study, 735 maize seeds from seven half-parent hybrid maize varieties were regarded as the research object. The maize seed images in the range of 900 ~ 1700nm were obtained by hyperspectral image acquisition system. The region of interest (ROI) of the embryo surface was selected, and the spectral reflectance of maize seeds was extracted. After Savitzky-Golay (SG) Smoothing pretreatment, Maximum Normalization (MN) pretreatment was performed. The 56 feature wavelengths were selected by Competitive Adaptive Reweighting Algorithm (CARS) and Successive Projection Algorithm (SPA). And the 56 wavelengths were mapped to high-dimensional space by high-dimensional feature mapping and then reconstructed into three-dimensional image features. A five-layer convolution neural network was used to identify three-dimensional image features, and nine (SG+MN)-(CARS+SPA)-CNN maize variety identification models were established by changing the input feature dimension and the depth factor size of the model layer.

**Results and Discussion:**

The results show that the maize variety classification model works best, when the input feature dimension is 768 and the layer depth factor d is 1.0. At this point, the model accuracy of the test set is 96.65% and the detection frame rate is1000 Fps/s in GPU environment, which can realize the rapid and effective non-destructive detection of maize varieties. This study provides a new idea for the rapid and accurate identification of maize seeds and seeds of other crops.

## 1 Introduction

As one of the three major food crops in the world, maize has a wide cultivation area, large yield and strong adaptability, which is of great strategic significance to the economic development and social stability of China (Feng et al., 2022). In the actual agricultural production process, cultivating suitable maize varieties is the crucial aspect to achieving high yields. Different maize seeds are easily confused due to various sorts and similar appearance, which brings great inconvenience to farmers in purchasing varieties and market supervision (Tu et al., 2021). Therefore, it is of great significance and application value to achieve rapid, accurate and efficient identification of maize varieties.

Traditional seed identification methods mainly include manual detection methods, chemical identification methods and so on, which have some defects such as intense subjectivity, great destructiveness and complex operation process. And they are challenging to meet the requirements of modern agriculture for non-destructive and rapid seed production (Wang et al., 2021; Wang and Wang 2021; Huang et al., 2022). Hyperspectral imaging technology combines the advantages of image and spectral technology, which can simultaneously reflect the image information and spectral information of external characteristics, internal physical structure and chemical composition of samples to be tested. So hyperspectral imaging technology is widely used in non-destructive testing research on crop seed varieties, quality and vitality (Wu et al., 2021; Wu et al., 2022; Yang et al., 2022). Huang et al. (2016b) used hyperspectral imaging technology to establish a PLS-SVM model to identify four different years of maize seeds, and the identification accuracy rate reached 94.4%. Fu et al. (2022) identified four maize varieties based on hyperspectral imaging technology, and the accuracy of the SSAE-CS-SVM model test set reached 95.81%. Wang et al. (2022) used hyperspectral imaging technology to establish a fusion model based on dual-band ratio image and texture features to realize efficient non-destructive identification of maize seeds of four different years, and the accuracy rate of prediction set was 97.5%. Chivasa et al. (2019) developed a PLS-DA model based on multi-temporal hyperspectral data and multivariate techniques to identify 25 maize varieties at specific phenological stages. Tu et al. (2022) used hyperspectral imaging technology combined with machine learning to realize non-destructive identification of 10 related maize varieties. Huang et al. (2016a) established the LS-SVM maize variety classification model based on hyperspectral imaging combined with spectral features and fusion with image features to identify 17 maize varieties with a test accuracy of more than 90%. Wu et al. (2016) collected hyperspectral image data of four maize varieties based on NIR hyperspectral technology, and established the SPA-PLS-DA classification model to realize non-destructive identification of maize varieties. The accuracy of the modeling set and prediction set reached 78.5% and 70.8%, respectively. Shao et al. (2019) collected hyperspectral images of three varieties of maize based on hyperspectral imaging system, screened characteristic bands by Boruta algorithm, and established a random forest classification model, with an accuracy rate of 78.30%. Sun M et al. (2022) modeled and analyzed wheat seeds of different seven years based on hyperspectral imaging technology, and predicted wheat seeds of other four years with an accuracy rate of 100%. Zhang et al. (2019) used hyperspectral image technology to obtain hyperspectral image spectral information of the wheat varieties mainly planted in Henan Province, and established PCA-SVM classification model, which identified three wheat varieties with an accuracy rate of over 95%. Sun Y et al. (2022) modeled and analyzed spectral information of moldy and non-moldy grains of different wheat varieties collected by hyperspectral imaging technology, and the prediction accuracy of SPA-SVM model for moldy grains was more than 98%.

Existing research shows that a large number of scholars at home and abroad have carried out research on crop seed variety identification, most of which are based on two methods: hyperspectral image information combined with deep learning and modeling based on spectral data. The method based on hyperspectral image mainly applies image features to identify seed varieties, which is suitable for identifying seed varieties with obvious shape and texture differences. While most seeds have no noticeable appearance differences, therefore, this method is difficult to be widely used in the identification of crop seed varieties in practice. The modeling methods based on spectral information are divided into two steps: feature band extraction and model building. The feature bands are mostly extracted by single extraction method such as SPA or CARS, which has some problems such as incomplete feature band extraction and lack of effective information. In addition, traditional machine learning models such as SVM, PLS and PCA are primarily used in modeling methods, which have the disadvantages of low accuracy and poor robustness. The convolutional neural network, as a kind of forward feedback network, can automatically learn the features in the image with higher accuracy and efficiency. Hybrid maize varieties are similar in appearance and not easily distinguishable, and subtle differences in the content of internal substances cause significant differences in yield, insect resistance, disease resistance, stress resistance and other indicators. Based on this situation, this paper was conducted with seven hybrid maize varieties as the research object, using SPA and CARS mixed feature band extraction method to improve the utilization rate of effective feature information, and building a convolution neural network (CNN) model based on data reshaping to achieve accurate identification of maize varieties.

## 2 Materials and methods

### 2.1 Test materials

**Figure 1:**
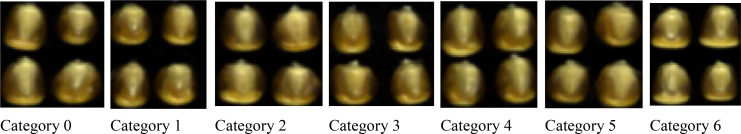
Maize seed sample.

The maize seed samples used in the experiment are all from the experimental field of Yanshi District, Luoyang City, Henan Province. Seven half-parent hybrid maize varieties with good appearance, uniform color and no mechanical damage were manually selected, marked as categories 0, 1, 2, 3, 4, 5 and 6, respectively. As shown in **Figure 1**, there were 105 seeds in each category, with a total of 735 test samples.

### 2.2 Instruments and equipment

**Figure 2:**
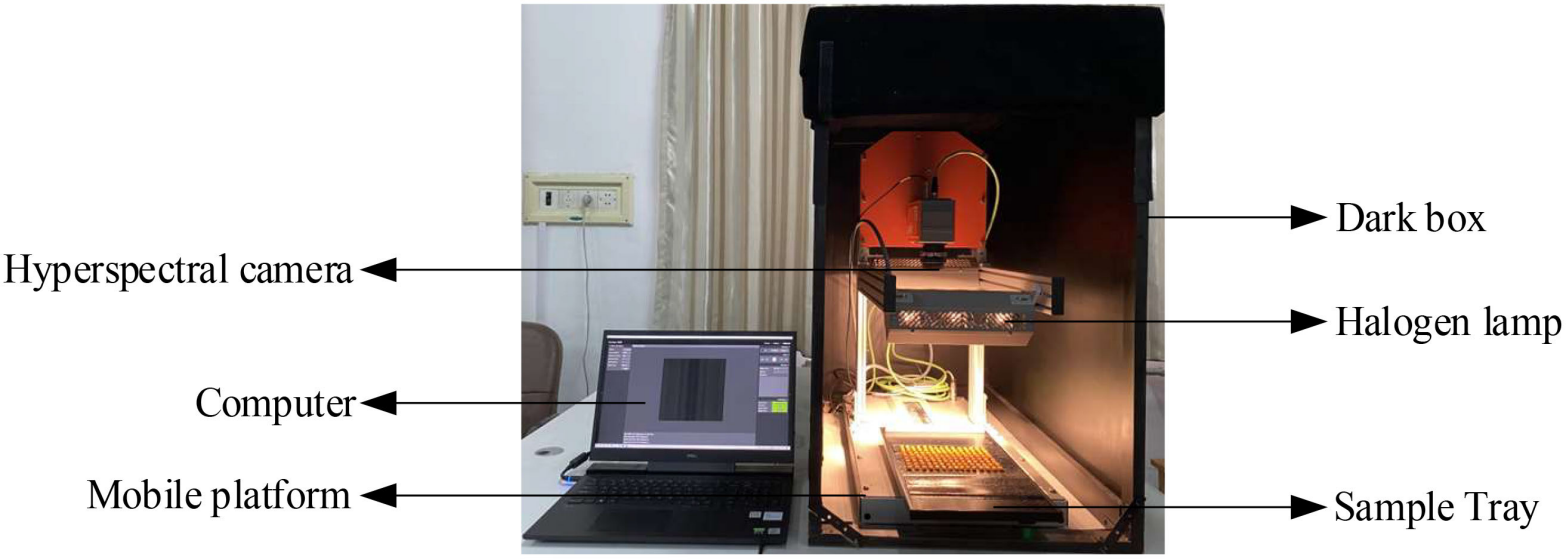
Hyperspectral image acquisition system.

The hyperspectral imaging system used in this test consists of a hyperspectral camera (SPECIM FX17, Finland), a computer (Dell), a mobile platform, a sample tray (40cm × 20cm) and six halogen lamps. To eliminate the effect of ambient light, maize seed spectra were collected in a dark box, as shown in **Figure 2**.

The parameters of the hyperspectral imaging system were set as follows: wavelength range is 900 ~ 1700nm, spectral resolution is 8nm, the number of bands is 224, spatial sampling resolution is 640px/line, exposure time is 8.5 ms, the frame rate is 50Hz and platform moving speed is 22.43 mm/s. Hyperspectral data of maize seeds were obtained by using Lumo Scanner software. The data analysis software is as follows: ENVI 5.3, The Unscrambler X10.4, Excel 2019, Origin 2018, MATLAB R2018b and so on.

### 2.3 Hyperspectral image acquisition and correction

**Figure 3:**
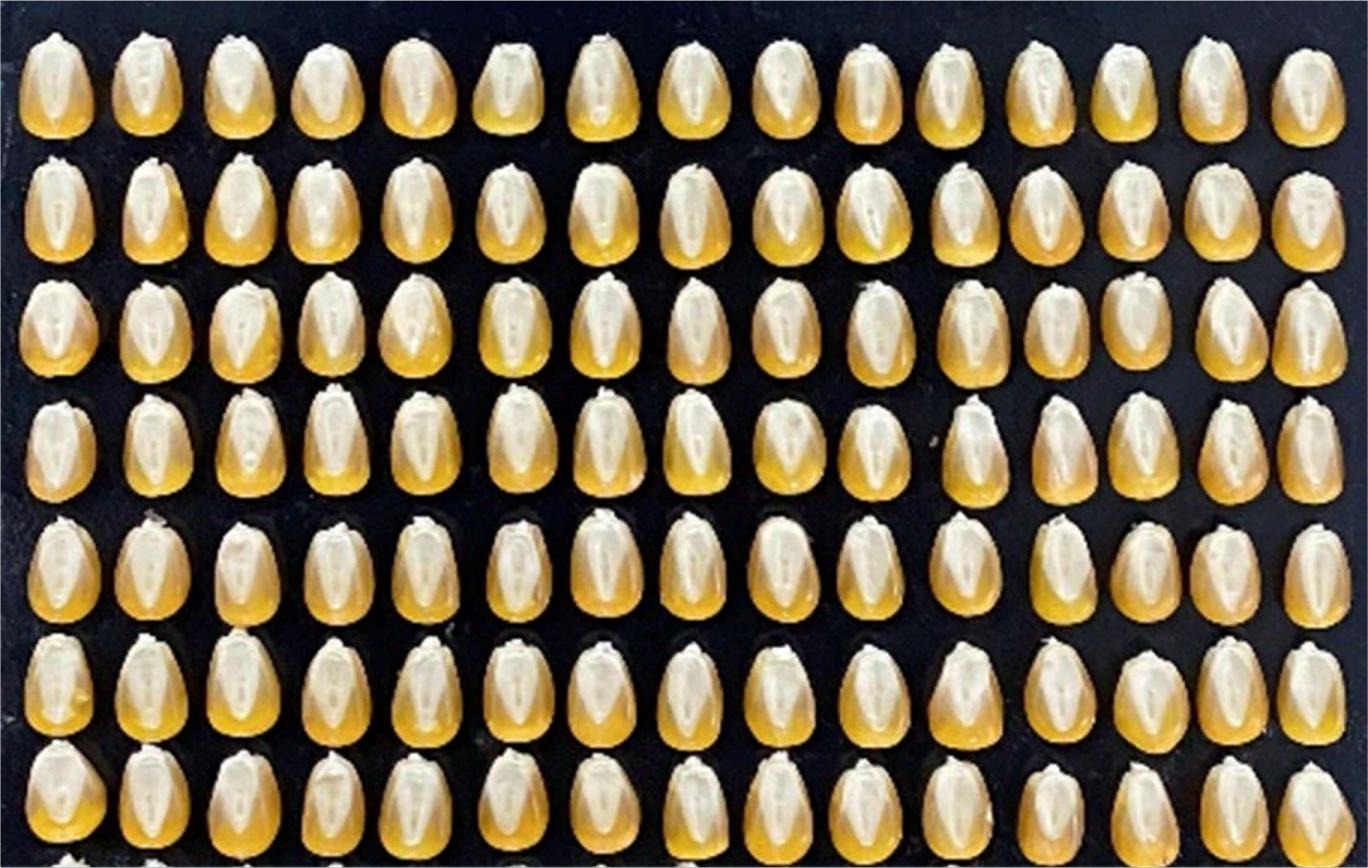
Schematic diagram of maize grain placement.

Maize seed embryos are rich in nutrients such as starch and protein, so the embryo surface image information of the sample was collected in this experiment (Wang et al., 2019). To ensure the accuracy of collected data and prevent maize seeds from moving on the mobile platform, the samples were placed on sticky black paper with their embryo face up. As shown in **Figure 3**, the images of 105 maize seeds of one variety were collected at a time, and a total of 735 single maize seed samples images were collected in the experiment.

Hyperspectral image is easily affected by nonlinear factors such as uneven distribution of light sources and dark current. To enhance the stability and reliability of the image, dark and white reference calibration images were used to correct the original hyperspectral image. The hyperspectral system was preheated for 30 minutes, the whiteboard (reflectivity 99%) was scanned and an all-white calibration image was recorded as *I*_w_, the lens cover was installed to collect all-black image which was recorded as *I*_d_, and finally the original image of maize sample was photographed and recorded as *I*_raw_. And the corrected image *I* is obtained by black-and-white correction with ENVI 5.3 software.


(1) $$I=\frac{I_{raw}-I_{d}}{I_{w}-I_{d}}$$

**Figure 4:**
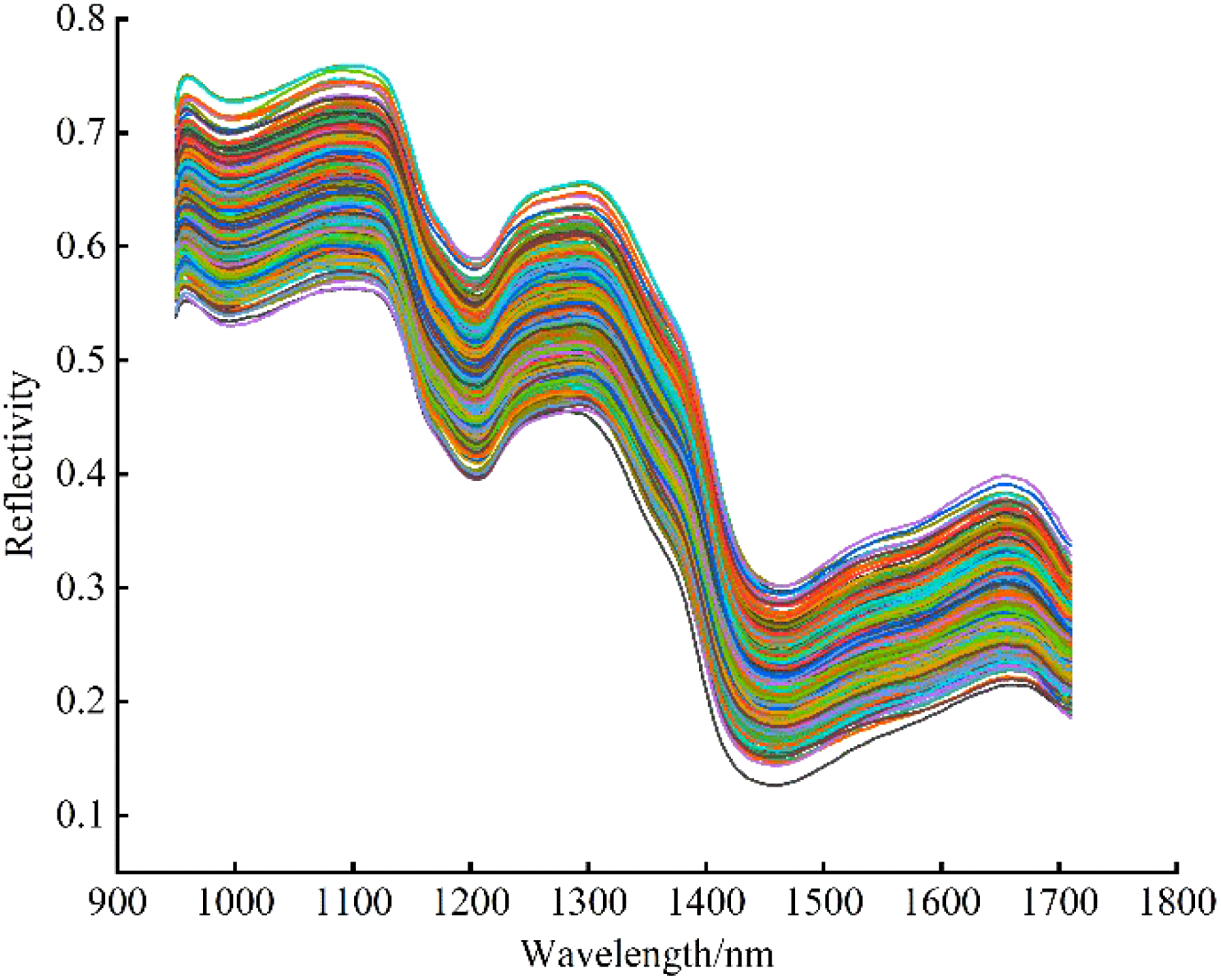
Reflectance curves of original spectrum.

After image correction, to reduce the influence of uneven distribution of chemical components in seeds, the largest possible rectangular ROI region was selected in the center of each seed sample by ENVI 5.3 software, and the average of the spectra of all pixel points within the ROI region was taken as the average spectrum of the sample (Feng et al., 2012). The original spectral average reflectance curve in the wavelength range of 935.61 ~ 1720.23 nm is shown in **Figure 4**. Due to both ends of the collected spectrum with low signal-to-noise ratio, the areas with considerable noise of spectral signal are eliminated, and the spectral data range of 949.43 ~ 1709.49 nm wavelength are selected for analysis and modeling.

### 2.4 Spectral preprocessing and feature wavelength selection

**Figure 5:**
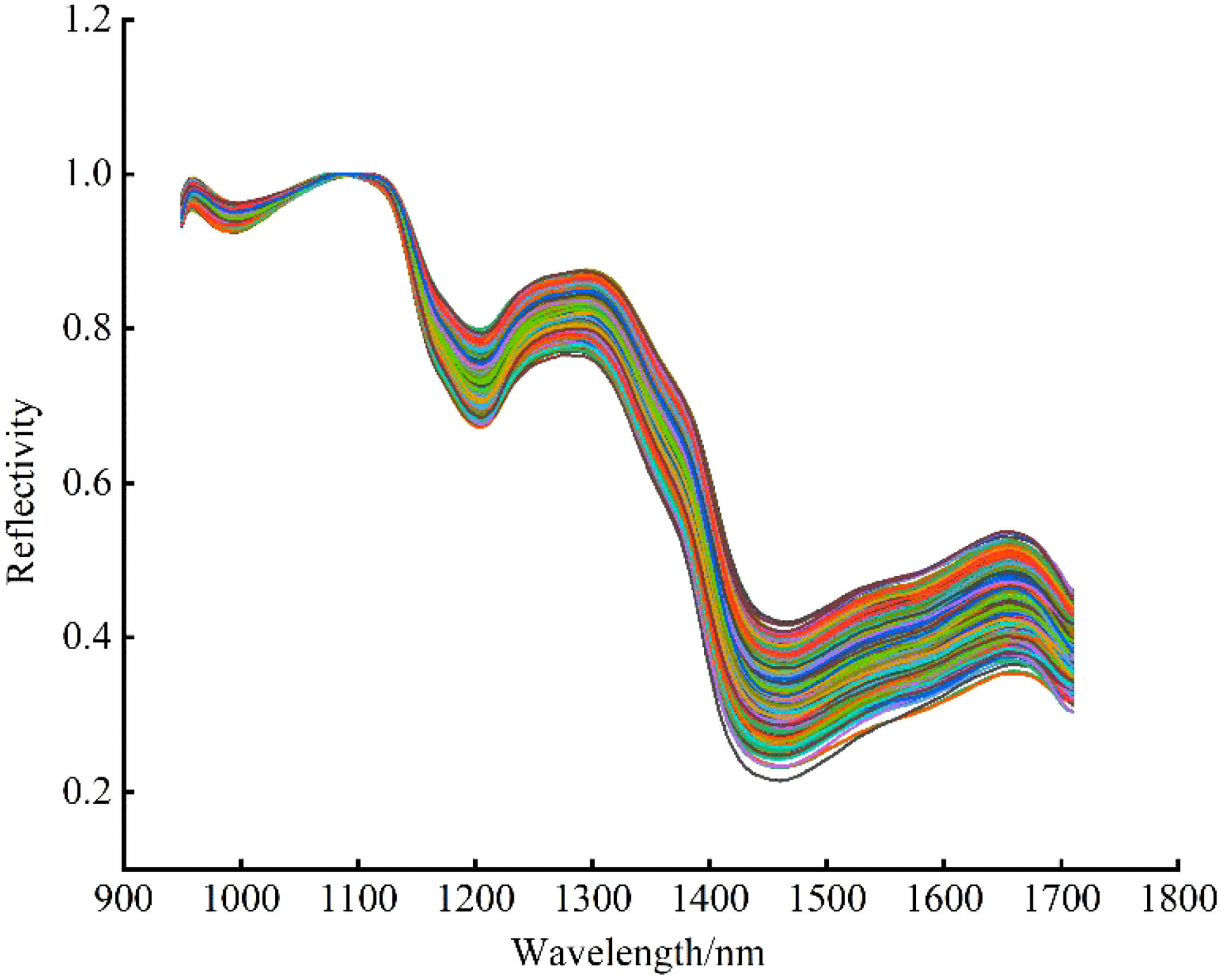
Spectral average reflectance curves after pretreatment.

The noise, background and other useless interference information mixed in the acquisition of spectra affected the accuracy and stability of spectral data analysis and modeling, so it is necessary to preprocess the data before modeling to reduce the interference of irrelevant information and improve the modeling accuracy. In this study, Savitzky-Golay Smoothing (SG) and Maximum Normalization (MN) were used to preprocess the data. Firstly, the number of smoothing points was set to 3, and the spectral data was pretreated by SG to improve the smoothness of the spectral curve. After that, the spectral data were mapped to the [0, 1] interval by MN, and the data unit restriction was removed to eliminate the errors caused by different magnitudes. The pretreated spectral average reflectivity curves are shown in **Figure 5**.

**Figure 6:**
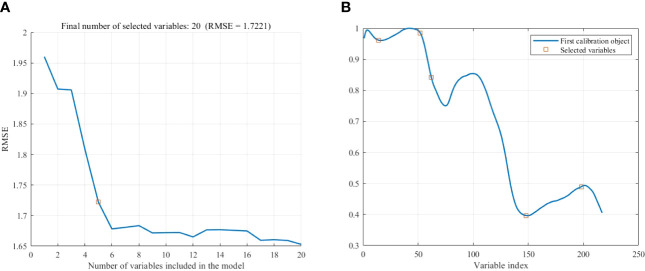
Extraction of feature wavelength by SPA. **(A)** Number of variables. **(B)** Location of variables.

The Successive projections algorithm (SPA) was used to extract the feature bands from the pretreated spectrum, the maximum number of wavelengths was set to 20, and five wavelength variables were extracted, as shown in **Figure 6**. As can be seen from **Figure 6A**, with the increase of the number of variables, the root mean square error (RMSE) value shows a trend of sharp drop at first and then slow down. When the number of variables is 5, the RMSE no longer decreases significantly and the RMSE value is 1.7221 at this time. After that, although the REMSE value decreases, too many dependent variables will increase the computation and complexity of the model, so five variables are selected as the final characteristic wavelengths.

**Figure 7:**
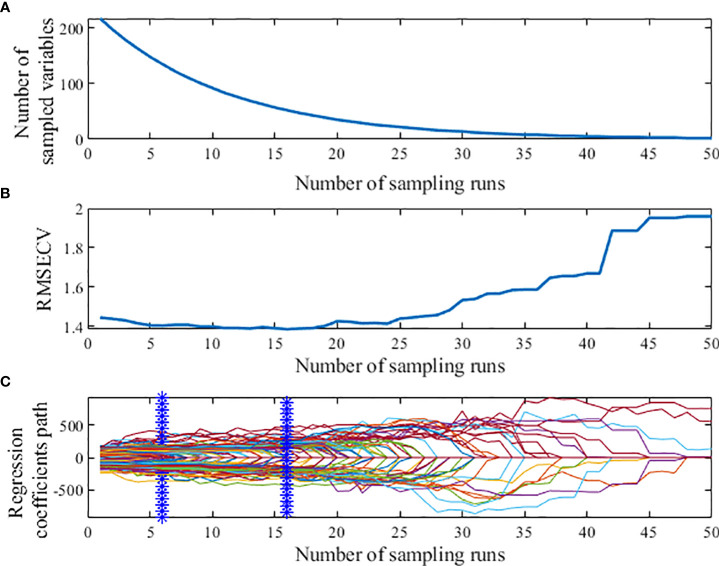
Process of extracting characteristic wavelength by CARS. **(A)** Number of preferred characteristic wavelength variables. **(B)** The root mean square error of cross-validation variation. **(C)** Regression coefficient path map.

When using Competitive adaptive reweighted sampling (CARS) to extract feature wavelengths, the 5-fold cross-validation method was selected, and the number of Monte Carlo samples was set to 50, as shown in **Figure 7**. **Figure 7A** shows that the number of CARS extracted feature wavelengths decreases sharply at first and then decreases slowly with the increase of sampling times, which shows the process from coarse to fine selection of feature wavelengths extracted by CARS. **Figure 7B** shows that the root mean square error of cross-validation (RMSECV) decreases slowly at the beginning of the iteration because the useless information bands are eliminated. And the RMSECV increases gradually after the 24th sampling, which indicates that the over-selection of feature wavelengths by CARS occurs after the 24th sampling and sensitive wavelength variables containing valid information are eliminated, resulting in the decrease of model prediction accuracy and the increase of RMSECV value; **Figure 7C** indicates that the RMSECV value is the smallest at the 6th and 16th sampling, when 52 characteristic wavelengths are extracted.

To solve the problem of missing effective information in the single extraction of feature variables by SPA and CARS, the feature wavelengths extracted by the two methods were taken as a concatenated set in this study, and a total of 56 feature wavelengths were preferentially selected.

### 2.5 Division of training set and test set

In this experiment, 735 samples were divided into training sets and test sets according to the ratio of 2: 1, where each category of training sets and test sets were 70 and 35 respectively. And seven categories of training sets and test sets were 490 and 245 respectively to analyze and calculate the discrimination accuracy of model training sets and test sets.

## 3 Model construction

### 3.1 Establishment of maize variety identification model

**Figure 8:**
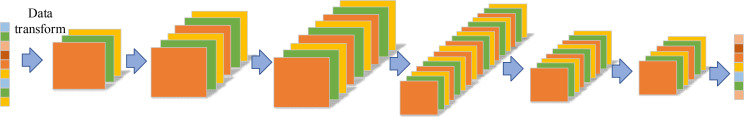
Overall network structure of CNN model.

To solve the problem that the Convolutional Neural Networks (CNN) cannot directly process the feature band data, the maize seed feature wavelength data was mapped to the high-dimensional features. Then the mapped feature wavelength data was reshaped into high-dimensional image features making the CNN processable for the reshaped data. The overall network structure is shown in **Figure 8**. The CNN model consists of three parts: data reconstruction, convolution layer extraction and result prediction. In the data reconstruction part, the feature bands of maize seeds are mapped into high-dimensional features with different sizes, and then reshaped into image shapes. Considering the dimension of maize seed characteristic band, it is not easy to build the convolution layers too deep to avoid overfitting and poor robustness of the model. Therefore, a 5-layer CNN maize variety discrimination model was constructed to improve the model generalization performance and reduce the redundancy effect of the model on spatial features. The specific model parameters are shown in **Table 1**. It can be seen from **Table 1** that the overall maize variety identification model is built by 3×3 standard convolution. To improve the spatial feature extraction effect of the model on maize seed feature bands, the sampling method of raising dimension first and then reducing dimension is adopted to fuse the features effectively. In order to explore the influence of model layers depth on the sampling effect of maize seed characteristic band, three common scaling factors (d), 1.25, 1.0 and 0.75 were used to scale the layers of maize variety identification model to different degrees. And the related parameters are listed in **Table 1**. In addition, to explore the influence of different high-dimensional feature resolutions on the adaptability of maize variety identification model and find the best adaptability resolution of the model, three different feature mapping relationships of 192, 768 and 3072 were used to generate three corresponding spatial feature resolutions of 8×8×3, 16×16×3 and 32×32×3. For the prediction part, adaptive maximum pooling operation and Softmax are mainly used to output the prediction results.

**Table 1:** Parameter index of CNN model.

Fom	Input			Operator	#out	Stride	Layer
0.75	32^2^×3	16^2^×3	8^2^×3	Conv2d 3×3	12	1	1
1.0	32^2^×3	16^2^×3	8^2^×3	Conv2d 3×3	16	1	
1.25	32^2^×3	16^2^×3	8^2^×3	Conv2d 3×3	20	1	
0.75	32^2^×12	16^2^×12	8^2^×12	Conv2d 3×3	24	1	2
1.0	32^2^×16	16^2^×16	8^2^×16	Conv2d 3×3	32	1	
1.25	32^2^×20	16^2^×20	8^2^×20	Conv2d 3×3	40	1	
0.75	32^2^×24	16²×24	8^2^×24	Conv2d 3×3	48	2	3
1.0	32^2^×32	16²×32	8^2^×32	Conv2d 3×3	64	2	
1.25	32^2^×40	16²×40	8^2^×40	Conv2d 3×3	80	2	
0.75	16²×48	8²×48	4²×48	Conv2d 3×3	24	1	4
1.0	16²×64	8²×64	4²×64	Conv2d 3×3	32	1	
1.25	16²×80	8²×80	4²×80	Conv2d 3×3	40	1	
0.75	16²×24	8²×24	4²×24	Conv2d 3×3	12	1	5
1.0	16²×32	8²×32	4²×32	Conv2d 3×3	16	1	
1.25	16²×40	8²×40	4²×40	Conv2d 3×3	20	1	

Fom represents depth factors of different sizes between model layers, Input represents 3D spatial feature matrices of different sizes, Operator represents corresponding convolution operations, out represents the size of feature maps output between model layers, Stride represents the step size of convolution kernel scanning, and Layer represents the names of convolution layers in different stages.

### 3.2 Building and training of model loss function

Because the maize variety classification model was a multi-category model, the Cross Entropy Loss function was used to regress training the maize variety identification model. The specific formula is shown in formula (2). In the formula, $y_{j}$ represents the unique thermal coding form corresponding to the actual category, and $o_{j}$ represents the probability that the network predicts a certain category.


(2) $$Loss=\sum_{j=1}^{q}y_{j}log\sum_{j=1}^{q}\exp\left(o_{j}\right)-\sum_{j=1}^{q}y_{j}o_{j}$$

The classification model of maize varieties is constructed with Pytorch framework. The hardware platform is Intel (R) Xeon (R) Silver 4210R, the main frequency is 3.5 GHz, and the memory is 32GB. The graphics card model is NIVIDIA GeForce RTX 2080Ti GPU, and the video memory is 16GB. The software platforms are Pycharm 2020.2, CUDNN 7.4. 1.5, Python 3.8 and Pytorch 1.2. The training period of epoch is set to 10000, and the initial learning rate is set to 0.01. The learning rate (lr) is adjusted periodically by LambdaLR algorithm, and the model parameters are optimized by SGD optimizer in one step to improve the training effect of the model.

### 3.3 Model evaluation index

To comprehensively evaluate the detection performance of maize variety classification model, training set accuracy (Train), test set accuracy (Test), frames per second (FPS), model weight (Weight), model computation (Flops), model parametric number (Params), Precision and Recall are used as evaluation indexes, and their specific calculation formulas are as follows.


(3) $$Precision=\frac{TP}{TP+FP}$$

(4) $$Recall=\frac{TP}{TP+FN}$$

(5) $$Accuracy=\frac{TP+TN}{TP+TN+FP+FN}$$

In the formula, TP represents the number of positive samples that the model prediction is consistent with the real label, FP represents the number of samples that the model prediction is inconsistent with the actual positive samples, FN represents the number of samples that the model prediction is inconsistent with the actual negative samples, and TN represents the number of samples that the model prediction is consistent with the actual negative samples.

## 4 Results and analysis

In this study, the maize variety identification model was trained and tested by 3-fold cross-validation to verify the applicability of maize variety identification model. When the characteristic dimension is 768, the specific results of cross-validation of three maize variety identification models, MVI_0.75_ D_1_, MVI_1.0_ D_1_ and MVI_1.25_ D_1_, are shown in **Table 2**. As can be seen from **Table 2**, the accuracy of training set and test set of MVI_1.0_D_1_ are 97.62% and 96.65% respectively, and the performance is the best. Compared with MVI_0.75_ D_1_ and MVI_1.25_ D_1_, the accuracy of MVI_1.0_D_1_ model test set is improved by 7.2% and 1.43%. The inference speeds of MVI_0.75_D_1_, MVI_1.0_D_1_ and MVI_1.25_D_1_ are 666Fps/s, 588Fps/s and 526Fps/s respectively in CPU mode and 1000Fps/s, 1000Fps/s and 909Fps/s in graphics card environment, which shows that the three models have real-time detection performance. Although MVI_0.75_D_1_, MVI_1. 0_D_1_ and MVI_1. 25_D_1_ are inputted the same spatial feature resolution the model detection results are significantly different in Weight, Params and Flops due to the influence of depth scaling factor of model layers. Weight, Params and Flops of MVI_0.75_D_1_ model are 2.33M, 70.35k and 286k respectively, which are the smallest among the three model. Weight, Params and Flops of MVI_0.75_D_1_ and MVI_1.25_D_1_ models are (4.05M, 6.25M), (90.73k, 116.86k) and (367k, 469k) respectively. Although there are obvious differences in the metrics of MVI_0.75_D_1_, MVI_1.0_D_1_ and MVI_1.25_D_1_, the parameters and calculation amount are still small and negligible compared with the classical CNN model. Therefore, it can be concluded that the detection effect of MVI_1.0_D_1_ is the best among MVI_0.75_D_1_, MVI_1.0_D_1_ and MVI_1.25_D_1_ three maize identification models.

**Table 2:** The model results of cross-validation when dimension is 768.

N	Model	Train/%	Test/%	Params	Flops	Weight	Fps_g_/s	Fps_c_/s
1	MVI_0.75_D_1_	91.73	86.12	70.35k	2.33M	286k	0.001	0.0015
2	MVI_0.75_D_1_	97.14	89.95	70.35k	2.33M	286k	0.001	0.0015
3	MVI_0.75_D_1_	95.55	88.52	70.35k	2.33M	286k	0.001	0.0015
1	MVI_1.0_D_1_	97.62	96.65	90.73k	4.05M	367k	0.001	0.0017
2	MVI_1.0_D_1_	99.84	95.69	90.73k	4.05M	367k	0.001	0.0017
3	MVI_1.0_D_1_	99.5	94.73	90.73k	4.05M	367k	0.001	0.0017
1	MVI_1.25_D_1_	98.73	92.82	116.86k	6.25M	469k	0.0011	0.0019
2	MVI_1.25_D_1_	99.84	95.22	116.86k	6.25M	469k	0.0011	0.0019
3	MVI_1.25_D_1_	99.84	93.78	116.86k	6.25M	469k	0.0011	0.0019

MVI_m_D_n_ represents different classification model of maize varieties. Among them, m represents the depth factor of model layer, m can be taken as 0.75, 1.0 and 1.25, n represents the input feature dimensions of different sizes, and n can be taken as 0, 1 and 2 respectively, which respectively represent the three states that the input feature dimensions are equal to 192, 768 and 3072. Train represents the accuracy of maize variety classification model in training set, and Test represents the accuracy of maize variety classification model in test set. Fps_g_ represents the frame detection speed in GPU environment, and Fps_c_ represents the frame detection speed in CPU environment.

To explore the influence of spatial feature dimension on the training results of maize variety identification model, the test results of maize variety identification model with two spatial feature dimensions 192 and 3072 were listed. The specific results are shown in **Tables 3** and **4**. The comparative analysis shows that the overall performance of the maize variety discrimination model MVI_1.25_D_0_ is better when the dimension is192 with the same width scaling factor d. In addition, it can be found from **Table 3** that when the width scaling factor d is 1.25, the accuracy rate of maize variety detection model in training set and test set is the best, which can reach 99.20% and 95.34% respectively. This reflects that when the feature space dimension is small, the maize variety identification model searches for effective features less effectively. Therefore, properly adjusting the depth scaling factor is helpful to improve the feature extraction ability and generalization performance of the model. According to the test results in **Tables 2**–**4**, it can be found that when the spatial feature resolution is enlarged only by improving the input feature dimension, the performance of the maize variety identification model is not better with a larger input feature dimension. Appropriate adjustment of the spatial feature dimension is helpful to improve the detection effect of the model. The best result is obtained when the dimension is 768, and the accuracy of maize variety identification model is improved obviously. In addition, the reasoning speed of CPU, Weight, Params, and Flops of maize variety identification model increased exponentially when the size of input feature dimension was changed, while the reasoning speed of GPU was basically stable at 1000Fps/s.

**Table 3:** The model results of cross-validation when dimension is 192.

N	Model	Train/%	Test/%	Params	Flops	Weight	Fps_g_/s	Fps_c_/s
1	MVI_0.75_D_0_	92.69	89.47	37.52k	582.04k	158k	0.001	0.001
2	MVI_0.75_D_0_	90.78	85.17	37.52k	582.04k	158k	0.001	0.001
3	MVI_0.75_D_0_	89.83	83.73	37.52k	582.04k	158k	0.001	0.001
1	MVI_1.0_D_0_	99.68	93.78	57.9k	1.01M	239k	0.001	0.0012
2	MVI_1.0_D_0_	94.28	86.60	57.9k	1.01M	239k	0.001	0.0012
3	MVI_1.0_D_0_	95.71	90.91	57.9k	1.01M	239k	0.001	0.0012
1	MVI_1.25_D_0_	98.57	92.83	84.03k	1.26M	341k	0.0011	0.0013
2	MVI_1.25_D_0_	99.20	95.34	84.03k	1.26M	341k	0.0011	0.0013
3	MVI_1.25_D_0_	99.52	94.26	84.03k	1.26M	341k	0.0011	0.0013

**Table 4:** The model results of cross-validation when dimension is 3072.

N	Model	Train/%	Test/%	Params	Flops	Weight	Fps_g_/s	Fps_c_/s
1	MVI_0.75_D_2_	81.08	75.60	201.68k	9.31M	800k	0.001	0.0026
2	MVI_0.75_D_2_	84.89	79.43	201.68k	9.31M	800k	0.001	0.0026
3	MVI_0.75_D_2_	80.45	75.60	201.68k	9.31M	800k	0.001	0.0026
1	MVI_1.0_D_2_	88.87	82.78	226.06k	16.29M	880k	0.001	0.0032
2	MVI_1.0_D_2_	86.49	83.73	226.06k	16.29M	880k	0.001	0.0032
3	MVI_1.0_D_2_	90.62	86.60	226.06k	16.29M	880k	0.001	0.0032
1	MVI_1.25_D_2_	98.57	91.39	248.19k	24.99M	982k	0.0011	0.0037
2	MVI_1.25_D_2_	99.20	87.56	248.19k	24.99M	982k	0.0011	0.0037
3	MVI_1.25_D_2_	99.52	92.34	248.19k	24.99M	982k	0.0011	0.0037

**Figure 9:**
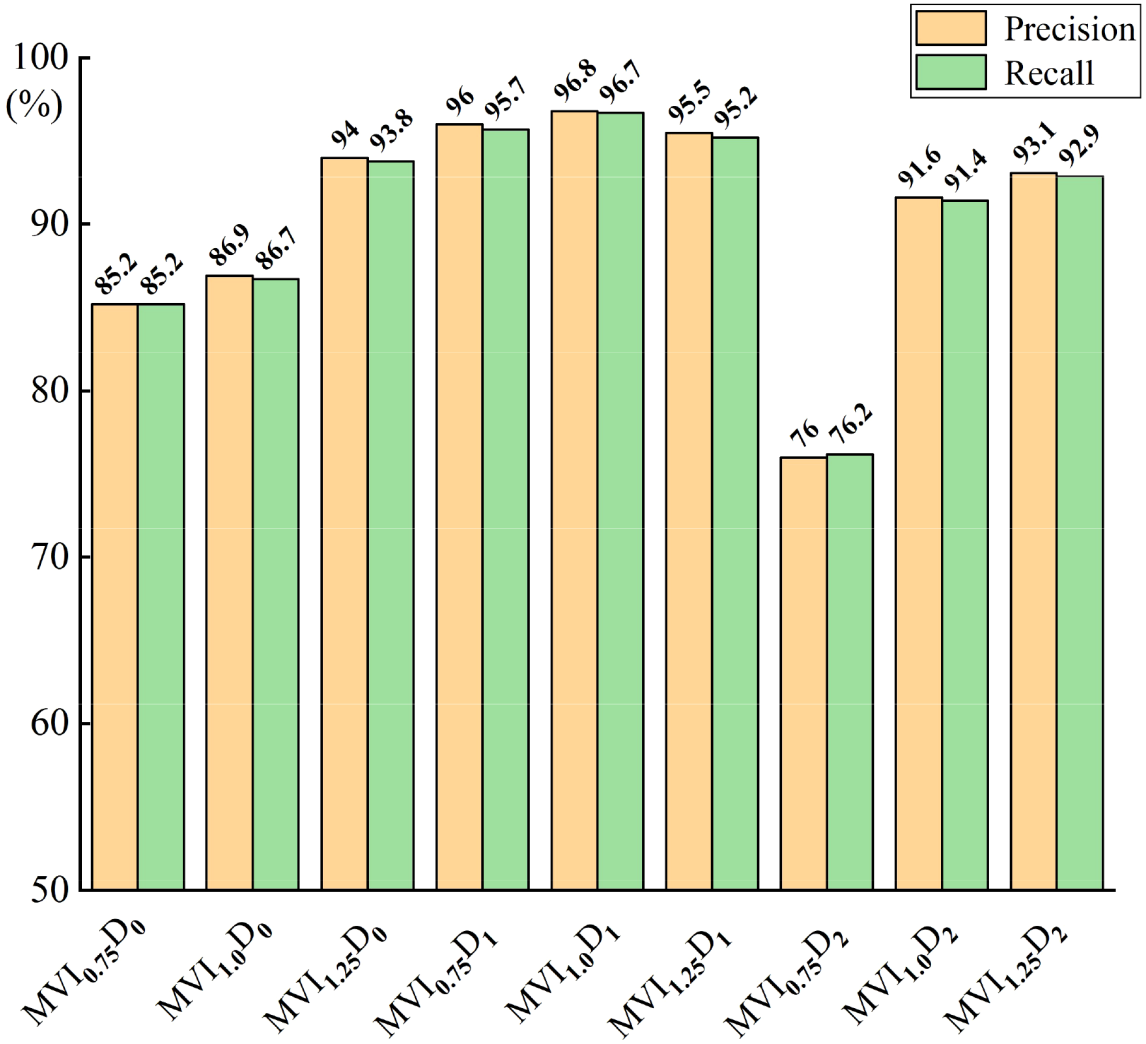
Recall and precision results of different models.

**Figure 10:**
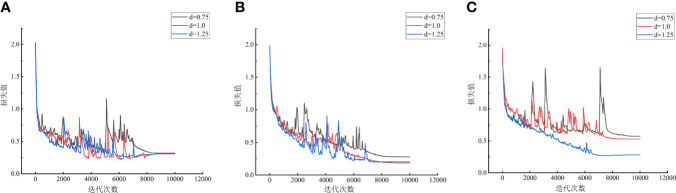
Loss curve of different models. **(A)** Dimension is192. **(B)** Dimension is 768. **(C)** Dimension is 3072.

In order to further explore the influence of input feature dimension and model layer depth on maize variety classification model, the Recall and Precision indexes of nine maize variety classification models were analyzed, as shown in **Figure 9**. It can be seen from **Figure 9** that the Recall and Precision of MVI_1.0_D_1_ model are the highest, respectively 96.7% and 96.8%, followed by maize variety classification models with the same characteristic dimension (dimension is768) and different model layer depths. The Recall and Precision of MVI_m_D_0_ model are more stable than MVI_m_D_2_, which also proves that the depth of model layer is not positively correlated with the performance of model classification. Appropriate adjustment of model layer depth is helpful to improve the effective extraction of spectral features of maize variety classification network. The variation of loss curves of nine models in 10000 iteration periods is shown in **Figure 10**. The loss curve of MVI_m_D_1_ model converges fastest with the increase of iterations and the overall fluctuation is slight. The loss curve of MVI_m_D_0_ model fluctuates more than that of MVI_m_D_1_, but the general convergence is faster. The loss curve of MVI_m_D_2_ is more divergent and the overall convergence is poorer with the increase of iterations, indicating that when the input dimension is 3072, it is easy to generate invalid feature redundancy, which is not conducive to the extraction of effective features by maize variety classification model.

**Figure 11:**
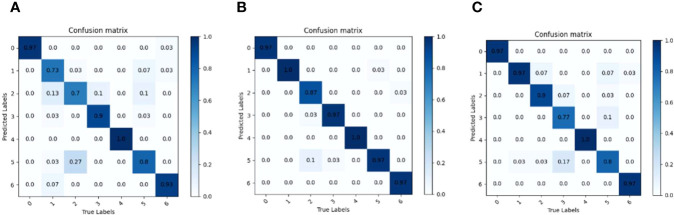
Model confusion matrix when layer depth scaling factor d is1. 0. **(A)** MVI_1.0_D_0_. **(B)** MVI_1.0_D_1_. **(C)** MVI_1.0_D_2_.

To explore the effects of MVI_m_D_0_, MVI_m_D_1_ and MVI_m_D_2_ series of maize variety classification models on seven kinds of maize seeds, three maize variety classification models (MVI_1.0_D_0_, MVI_1.0_D_1_ and MVI_1.0_D_2_) with layer depth scaling factor d=1. 0 were selected to test the test set, and the correlation confusion matrix was drawn by comparing the predicted results with the actual results, as shown in **Figure 11**. It can be seen from **Figure 11B** that MVI_1.0_D_1_ is the best in classifying seven maize seeds and there are no misidentifications and omissions in category 1 and category 4. Although the MVI_1.0_D_1_ model shows misrecognition among categories 1, 3, 5 and 6, the misidentification rate is lower compared with the confusion matrix results of MVI_1.0_D_0_ and MVI_1.0_D_2_, and MVI_1.0_D_1_ only misidentifies category 0 without misidentification. Compared with MVI_1.0_D_1_, MVI_1.0_D_0_ and MVI_1.0_D_2_ show more misidentification and the model classifiers are unbalanced.

In this study, seven hybrid maize varieties were taken as the research objects, and the effects of different input feature dimensions and model layer depth on the performance of the maize variety classification model were discussed emphatically. The results showed that the maize variety classification model performs better when the input feature dimension is 768 and worse when the input feature dimension is 3072. This phenomenon may be attributed to the redundant and invalid features easily produced by the higher feature dimension, which indirectly affects the classification effect of the maize variety classification model. Therefore, changing the dimension of input features can effectively improve the ability of extracting effective spectral features of maize variety classification model. In addition, the effect of model layer depth on the performance of maize variety classification model was also discussed in this study. From the results, it can be found that there is no positive correlation between the performance of maize variety classification model and the layer depth of the model. When the layer depth factor d is 1.25, the performance of the maize variety classification model is slightly lower than that when d is 1. 0, so it is most appropriate to set the layer depth factor d as 1. 0. Due to the small sample size, more sample data will be collected in the future to further validate the maize classification model whether the method of identifying maize varieties by mapping characteristic bands to high-dimensional spatial features is feasible.

## 5 Conclusion

(1) To solve the problem of less effective feature bands and lack of information by single feature variable extraction method, 56 feature bands are selected by combining SPA and CARS in this study.
(2) To solve the problems of poor effect and slow speed of traditional machine learning method in maize classification, a high-dimensional feature mapping method is adopted to reshape the extracted feature bands into three-dimensional image features after mapping them to a high-dimensional space. And a five-layer convolution neural network is constructed to identify three-dimensional image features.
(3) At the same time, the influence of the size of the input feature dimension and the depth of the model layer on the performance of the maize variety model are discussed in this study. The test results show that when the dimension of the input feature dimension is 768 and the depth factor of the layer is 1.0, the performance of maize variety classification model is the best. And the accuracy of the test set is 96.65%, and the detection frame rate is 1000Fps/s in GPU environment, which can realize rapid and effective non-destructive detection of maize varieties.

## Data availability statement

The raw data supporting the conclusions of this article will be made available by the authors, without undue reservation.

## Author contributions

FZ: Funding acquisition, Investigation, Project administration,Resources, Supervision, Writing – review & editing. FYZ: Data curation, Formal Analysis, Writing – original draft, Writing –review & editing. SQW: Formal Analysis, Methodology, Software, Supervision, Writing – original draft. LTL: Investigation, Resources, Supervision, Writing –review & editing. QL: Data curation, Funding acquisition, Investigation, Resources, Supervision, Writing – review & editing. SLF: Investigation, Resources, Supervision, Writing – review & editing. XYW: Supervision, Writing – review & editing. QFL: Investigation, Resources, Writing – review & editing. YKZ: Investigation, Supervision, Writing – review & editing.
